# Testing a Short Nuclear Marker for Inferring Staphylinid Beetle Diversity in an African Tropical Rain Forest

**DOI:** 10.1371/journal.pone.0018101

**Published:** 2011-03-31

**Authors:** Birthe Thormann, Michael J. Raupach, Thomas Wagner, Johann W. Wägele, Marcell K. Peters

**Affiliations:** 1 Zoological Research Museum Alexander Koenig, Bonn, Germany; 2 German Centre for Marine Biodiversity Research, Research Institute Senckenberg, Wilhelmshaven, Germany; 3 Abteilung Biologie, Institut für Integrierte Naturwissenschaften, Universität Koblenz – Landau, Koblenz, Germany; 4 Department of Animal Ecology and Tropical Biology, Biocenter, University of Würzburg, Am Hubland, Würzburg, Germany; Field Museum of Natural History, United States of America

## Abstract

**Background:**

The use of DNA based methods for assessing biodiversity has become increasingly common during the last years. Especially in speciose biomes as tropical rain forests and/or in hyperdiverse or understudied taxa they may efficiently complement morphological approaches. The most successful molecular approach in this field is DNA barcoding based on cytochrome *c* oxidase I (COI) marker, but other markers are used as well. Whereas most studies aim at identifying or describing species, there are only few attempts to use DNA markers for inventorying all animal species found in environmental samples to describe variations of biodiversity patterns.

**Methodology/Principal Findings:**

In this study, an analysis of the nuclear D3 region of the 28S rRNA gene to delimit species-like units is compared to results based on distinction of morphospecies. Data derived from both approaches are used to assess diversity and composition of staphylinid beetle communities of a Guineo-Congolian rain forest in Kenya. Beetles were collected with a standardized sampling design across six transects in primary and secondary forests using pitfall traps. Sequences could be obtained of 99% of all individuals. In total, 76 molecular operational taxonomic units (MOTUs) were found in contrast to 70 discernible morphospecies. Despite this difference both approaches revealed highly similar biodiversity patterns, with species richness being equal in primary and secondary forests, but with divergent species communities in different habitats. The D3-MOTU approach proved to be an efficient tool for biodiversity analyses.

**Conclusions/Significance:**

Our data illustrate that the use of MOTUs as a proxy for species can provide an alternative to morphospecies identification for the analysis of changes in community structure of hyperdiverse insect taxa. The efficient amplification of the D3-marker and the ability of the D3-MOTUs to reveal similar biodiversity patterns as analyses of morphospecies recommend its use in future molecular studies on biodiversity.

## Introduction

Tropical rain forests harbor the most species-rich animal communities on earth [1]. In this biome, as in other terrestrial ecosystems, insects make up the largest part of the diversity, also constituting the overwhelming majority of animal biomass and of numbers of individuals [2], [3]. While tropical rain forests are being rapidly destroyed, the consequences for most insect taxa are still little understood and biotic changes of communities over time have scarcely been monitored. This is mostly due to a lack of taxonomic expertise for many taxa and the large time effort and monetary costs of sample processing and species identification for several hundred to thousands of specimens, which typically occur in biodiversity surveys of insects.

During the last years a variety of new molecular genetic approaches to taxa recognition have been established to circumvent the difficulties of traditional taxonomy (e.g. [4]–[6]). These new technological approaches may accelerate biodiversity inventories, may help in documenting the presence of insect species before they become extinct, and may offer a feasible way to monitor extremely abundant and diverse insect groups. The most widely used molecular genetic approach to identify organisms with the aim of providing a reliable, cost-effective and accessible solution to the current problems of species identification and delimitation is DNA barcoding [7]. During the last years barcodes have been tested for different questions and in a variety of taxa (e.g. [8]–[14]). However, most studies aimed at identifying (e.g. [15]) or delimiting species (e.g. [11]), while to date only few studies applied those methods to answer ecological questions (e.g. [16]–[18]). In particular, the suitability of molecular approaches to reveal biodiversity patterns of (non-microbial) animal communities from standardized monitoring samples has scarcely been tested (e.g. [6], [19], [20]).

Besides the widely used mitochondrial cytochrome *c* oxidase I (COI) gene, a variety of markers have been used to identify or delimit species or species-like units. For example, the variable loops in nuclear ribosomal DNA (rDNA) sequences, in particular those of the large subunit rRNA (LSU) which are not inherited maternally and avoid problems of mitochondrial markers (introgression, pseudogenes), have been proposed as a reasonable alternative to COI [21]. Although ribosomal genes are generally considered to be highly conserved, Sonnenberg et al. [22] have shown that the D1 and D2 expansion segments of the 28S rRNA hold fast evolving and variable regions which can be used for identification in a wide variety of species across a broad range of various Metazoan taxa and can resolve even very closely related species. Highly conserved regions which flank the variable regions allow the use of ‘universal’ primers (working for most metazoan taxa, vertebrates and invertebrates as well) [22], a necessary feature for the identification of specimens whose taxonomic belonging is not known *a priori*. Beside this, nuclear ribosomal genes occur in tandem repeats, making them easily retrievable also from very small or partially degraded samples.

Here we test a molecular approach of assessing biodiversity patterns in one of the most speciose and understudied animal groups, the Staphylinidae (rove beetles), of a Guineo-Congolian rain forest. With 55,440 described species [23], a worldwide distribution, and a very wide range of habitat use, Staphylinidae Latreille, 1802 (Coleoptera, Staphyliniformia) represent one of the largest and most successful families of Coleoptera [24], [25]. In many ecosystems they are a major component of the arthropod fauna, and they are of high functional importance as predators and scavengers, as well as partners in several symbioses with other organisms [25], [26]. Due to often very subtle morphological difference among species and their minute size, morphological delimitation of staphylinid beetle species is challenging and requires high taxonomic expertise. For species-level identification dissection of the genitalia of specimens is often necessary [26], which is, however, not feasible in large-scale biodiversity studies where often hundreds or thousands of specimens accumulate. So these two features of the group, (i) their belonging to a highly diverse and abundant but understudied group, and (ii) their subtle and challenging morphological features, make the taxon ideally suited for testing a DNA based approach of assessing biodiversity.

In the present study, the D3 fragment of the nuclear 28S ribosomal DNA with a length of ∼180 bp was tested as molecular marker. In contrast to the widely used mitochondrial COI gene, it is not inherited maternally. It is, however, less variable and may not always contain species-specific substitutions. We use the D3-marker to delimit molecular operational taxonomic units (MOTUs), groups of sequences that represent working units that do by proxy - but not necessarily exactly - correspond to real species [27], [28]. We compare biodiversity patterns derived from the MOTU data set to those derived from a rapid morphological assessment in order to test the usefulness of the D3-marker for delimiting meaningful, species-like biodiversity units as surrogates for species and to assess biodiversity patterns. In particular, we asked the following questions: (i) Can the D3 fragment be successfully obtained from all specimens of the sampled staphylinid beetle community and can it therefore be considered to be a ‘universal marker’ for this group? (ii) Does the molecular approach reveal the same number and density of species and similar species community differences as a morphology-based biodiversity assessment? (iii) Are differences in diversity and community composition comparing primary and secondary forests described in the same way by the molecular approach as in a morphological assessment?

## Materials and Methods

### (a) Sampling

**Figure 1:**
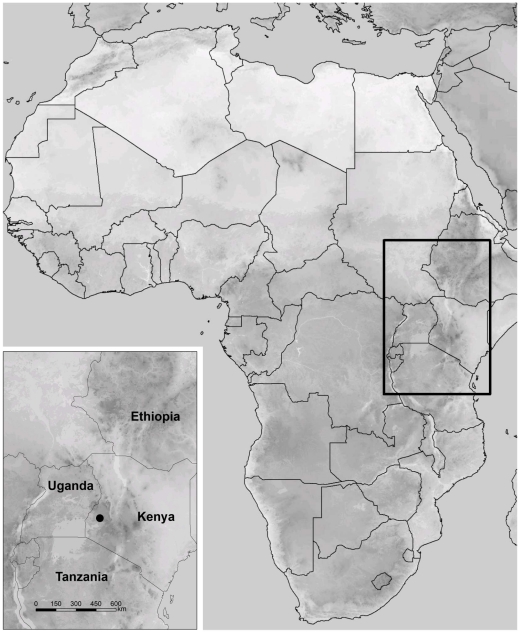
Map of Africa. Location of Kenya and location of the study area (Kakamega Forest) within Kenya.

Sampling was conducted during the rainy season in April and May 2008 in the Kakamega Forest, a tropical rain forest situated in the Western Province of Kenya, about 50 km north-east of Lake Victoria (00°10′N–00°21′N, 34°47′E–34°58′E; Fig. 1). It is located between 1460 and 1765 m above sea-level. Due to its equatorial location, the forest exhibits a tropical daytime climate with a distinct daily variation in temperature between a minimum of about 13°C and a maximum of 34°C. Mean annual precipitation is 1947 mm, concentrated in two rainy seasons per year.

Flora and fauna of the Kakamega Forest, which is considered to be the easternmost relict of the Guineo-Congolian rain forest area, contains elements of both the west and central African lowland rain forests, as well as species of the Afro-montane forests of East Africa (e.g. [29], [30]). Kakamega Forest and its rich biodiversity are suffering an enormous anthropogenic pressure due to its location in one of the most densely populated rural regions worldwide in combination with high poverty. The long history of disturbance and exploitation lead to a high degree of fragmentation and degradation. Today the main forest block covers only 8245 ha comprising a heterogeneous mixture of different succession stages such as disturbed primary forest, secondary forest, clearings, and tea and timber plantations creating a mosaic-like structure of different habitats.

Sampling took place along six 200 m long transects, whereof three were placed in primary forest and three in secondary forest habitat. Primary forests show a high canopy of 25–30 m, dense undergrowth dominated by *Dracaena fragrans*, and in most parts a species-rich layer of medium sized trees [31]. However, due to high anthropogenic pressure certain tree species were selectively logged in the last decades so that the forest is not in a pristine state [31]. Secondary forests are habitats with lower vegetation heights of 5–15 m. They contain pioneer species and exotic elements, often without a closed canopy layer. In both habitat types the forest floor was covered by much leaf litter. To avoid spatial autocorrelation, the minimum distance between transects was 254 m and the transect next to a primary forest transect was always a secondary forest transect and vice versa. Transects were situated at least 50 m inside the habitat to avoid edge effects (see supplementary material: Fig. S1, Table S1 for coordinates and a map showing the locations of transects).

Staphylinidae were collected by pitfall trapping following the methodology described by Bestelmeyer [32]. Pitfall trapping is a widely used method for catching leaf litter-dwelling arthropods and has also been successfully used for collecting staphylinid beetles (e.g. [33], [34]). Leaf litter sifting, as other effective method for collecting most staphylinid taxa was not carried out due to its high efforts of time and labor in order to fit into the concept of an efficient and rapid biodiversity assessment.

On each transect ten pitfall traps (ø 7.5 cm plastic cups with a 12×12 cm rain cover at a height of 12 cm) were placed, 20 m apart from each other. To obtain the highest possible conservation of DNA-material, pitfall traps were filled with 100 ml of 95% ethanol and recollected and cleared after two days. For each trap staphylinid beetles were separated from other animals under a microscope and preserved in 2 ml tubes filled with absolute ethanol. Finally, all staphylinid beetles of each particular trap were dried on a tissue, examined through a stereomicroscope and categorized into morphospecies. From each morphospecies of each trap one specimen served as a sample for the genetic analysis as well as for the morphological classification. For the purpose of the latter one, each beetle was mounted on a card and labeled.

### (b) Morphological assignment

Morphological categorization of the specimens into morphospecies was conducted by one of the authors (T.W.) on the basis of external morphology but without genital dissection, or the use of identification literature. This ‘parataxonomic’ sorting has become one of the most efficient approaches for the study of biodiversity in tropical ecosystems available to date [35], [36]. Often it is the only feasible method to handle the huge amount of insect specimens typically sampled in biodiversity studies, where a ‘true’ taxonomic identification, which mostly would involve dissection and the time-intensive use of identification keys, would be impossible (e.g. [35]–[37]). Parataxonomic units (e.g. morphospecies) seem to approximate species sufficiently well to be used in ecological assessments for terrestrial invertebrates [38], [39]. However, a low accuracy and sorting errors that may cause problems in analyses and testability of results is criticized by some authors (e.g. [40], [41]). Although usually morphospecies just receive numbers, in our study they were in addition assigned to genera. All staphylinid beetles were deposited in the Coleoptera collection at the Zoological Research Museum Alexander Koenig (ZFMK) (see supplementary material: Table S2), where they are available for more detailed taxonomic revision, which is desirable as ultimately a taxonomic identification should be embedded in biodiversity studies. So genetic data is not dissociated from the individual, but every sequence can be assigned to its specimen of origin and vice versa.

### (c) DNA extraction, amplification and sequencing

Total genomic DNA was extracted from up to three dissected single legs of each specimen, using the Qiagen DNeasy® Blood&Tissue Kit, following the manufacturers' protocol. DNA was eluted with 100 µl buffer AE; this step was repeated once to maximize yield. The preservation of the whole animal permitted the employment of the same individual for molecular analyses as well as for morphological assignment and collection voucher.

For amplification of the about 180 bp long D3 fragment and parts of adjacent regions the primers CD3F and CD3R (5′- GGA CCC GTC TTG AAA CAC -3′ and 5′- GCA TAG TTC ACC ATC TTT C -3′; [42]), and the Qiagen® Multiplex PCR Kit were used. Amplification reactions were carried out in a 20 µl volume containing 10 µl QIAGEN Multiplex PCR Mastermix, 2 µl Q-Solution, 1.6 µl of each primer (both 10 pmol/µl), and 2 µl DNA template, and filled up to 20 µl with sterile H_2_O. The PCR temperature profile consisted of an initial denaturation at 95° (15 min), followed by 35 cycles at 94° (35 s, denaturation), 52° (90 s, annealing), 72° (90 s, extension), and a final extension at 72° (10 min).

Products were checked by electrophoresis on a 1.5% agarose gel containing ethidium bromide. Successfully amplified DNA fragments were purified using the Qiagen QIAquick® PCR Purification Kit following the manufacturers' protocol. Purified PCR product was eluted with 35 µl elution buffer EB.

Samples were bidirectionally sequenced by a commercial company (Macrogen Inc., Seoul, Republic of Korea; http://www.macrogen.com) using PCR primers. BLAST searches [43] were performed to confirm the identity of the new sequences. Sequences of all MOTUs are deposited in GenBank; for MOTUs which contain more than one morphospecies, additional sequences were submitted (accession numbers HM583881–HM583967).

### (d) Sequence analyses and statistical analyses

Contigs were assembled with Lasergene SeqMan II (DNA-Star) 4.03 and aligned using MUSCLE 3.6 [44] (default settings were retained except maximum number of iterations (maxiters) = 1000). Identical sequences have been removed. Subsequently, a Neighbor-Joining-Tree (NJ-Tree) based on p-distances [45] was generated using PAUP* 4.0b10 [46].

According to differences in the sequences, specimens were categorized into MOTUs (molecular taxonomic operational units), using a difference of one basepair (bp) as threshold to assign a specimen to a different MOTU. The goal was to compare the resolution obtained with the D3-marker with the diversity estimated from morphospecies. The difference of one single basepair to delimit MOTUs is based on the results of a previous extensive study of the taxonomically well understood central European ground beetles (Coleoptera: Carabidae). Within this group a difference of one single basepair of the D3 sequence invariably indicated different morphologically valid species [42]. However, when divergent populations have very recent origins or still hybridize, the use of rDNA sequences for species identification is as ambiguous as the attempt to discern species morphologically: after the initial split, divergent populations will share alleles and mutations in slowly evolving genes [21]. However, as closely related species usually have distinct distributional ranges (due to competitive exclusion of species sharing similar ecological niches and because speciation mostly occurs in allopatry; [47]), few sister species pairs should be expected to co-occur in a local area. Therefore, we assume the potential problem of the D3-marker to give too conservative estimates of species diversity to be rather small for surveys conducted on a local level, e.g. on the level of transects in a small study area like the Kakamega Forest.

Species accumulation curves were used to visualize the increase in total species diversity in relation to the number of sampled pitfall traps and to check the completeness of our faunal survey. The expected total number of species was estimated using the first-order Jackknife estimator, which showed best performance in both simulated and real data when sampling effort is low [48].

The congruence of morphological and molecular methods on assessing species richness (total number of morphospecies or MOTUs per transect respectively) and species density (mean number of morphospecies or MOTUs respectively found in each pitfall trap on transects) was compared by paired t-tests. Dissimilarity of species communities among transects were calculated using the Bray-Curtis dissimilarity index and matrices derived from two methodological approaches were compared using Mantel tests.

In order to analyze the utility of the two methodological approaches for identifying differences in biodiversity patterns between primary and secondary forests, the following analyses were done separately for the molecular data and the morphological data: Welch-two-sample t-tests were used to compare mean richness and density of taxa between habitats.

Detrended correspondence analysis (DCA) was used to analyze and visualize the effect of habitat on species composition. Subsequently, habitat was fitted as an explaining variable to the first two DCA axes and its significance in structuring beetle communities was tested by permutation tests based on 1,000 replications.

All statistical analyses and visualizations were carried out with R 2.6.0, except the barplots in Fig. 3 which were plotted with Microsoft Office Excel 2007.

## Results

In total, 1,517 staphylinid specimens were sampled which were assigned to 70 morphospecies. After sorting to morphospecies, for each trap one specimen of each morphospecies was analyzed, summing up to a total of 425 individuals. Amplification and sequencing of the D3 fragment was successful for 421 specimens (99.1%). In total, 76 MOTUs were identified by using the 1 bp threshold (see supplementary material Fig. S2). A threshold of 2 or 3 bp to delimit MOTUs changed the number of MOTUs only slightly (70, respectively 63 MOTUs).

**Figure 2:**
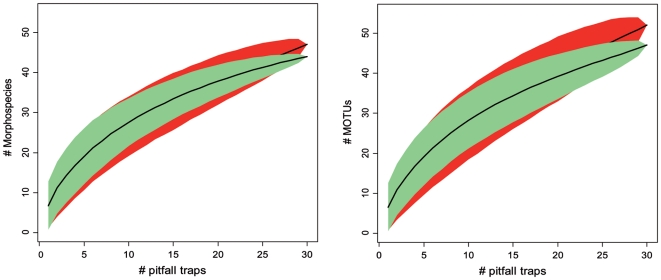
Species accumulation curves of primary and secondary forest. A. Increase in the number of morphospecies with increasing number of analyzed pitfall trap samples for primary (green) and secondary forest (red). B. Increase in the number of MOTUs with increasing number of analyzed pitfall trap samples for primary (green) and secondary forest (red). Colored polygons indicate 95% confidence intervals.

Species accumulation curves were nearly identical for both approaches and did not reach saturation (Fig. 2, see also supplementary material Fig. S2), indicating that by further sampling additional species would probably have been found. The expected total number of morphospecies estimated with the first-order Jackknife estimator was 100 (SE = 14) while the expected total number of MOTUs was 114 (SE = 20), i.e. the species communities were estimated to be similarly rich by the molecular and the morphological approach.

In 30 taxa the identification of morphospecies and MOTUs was exactly consistent, which means that one MOTU consisted exclusively of members of one morphospecies and contained all members of this morphospecies and vice versa. In total, 25 of the 421 analyzed individuals are involved in ‘splittings’ (one MOTU includes members of two or three different morphospecies), 32 are involved in ‘lumpings’ (one morphospecies includes members of two or three different MOTUs).

**Figure 3:**
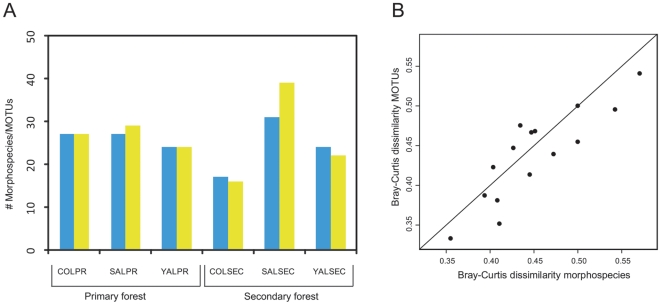
Species richness and community dissimilarities inferred by molecular and morphological approach. A. Species richness on transects based on the morphological (blue) and molecular genetic approach (yellow). B. Bray-Curtis community dissimilarities among six transects (resulting in a total of 15 comparisons) based on molecular data (MOTUs) and morphological data (Morphospecies). The line shows the ideal condition of total congruence of the two methodologies.

However, when comparing species richness on transects, no significant difference was found between the morphological and the molecular approach (paired t-test, mean difference = 1.17, t = −0.79, df = 5, p = 0.46) (Fig. 3A). The transects with the highest and the lowest species richness were the same in both methods. Likewise, similarity of species communities was estimated in a similar way by both methods: Bray-Curtis dissimilarity reached from 0.33 to 0.54 when using the molecular approach and from 0.36 to 0.57 in case of the morphological approach. Dissimilarity matrices were strongly correlated between the two approaches (Mantel-test: r = 0.86, p<0.001), i.e. pairs of transects which showed high overlap in species communities in the molecular approach were also highly similar using the morphological approach (Fig. 3B). The only difference of results of the two methods was found for species density, which was slightly higher when relying on morphological data than when using the MOTU data (paired t-test, mean difference = 0.15, t = 4.39, df = 5, p<0.01) (see supplementary material Fig. S3).

**Figure 4:**
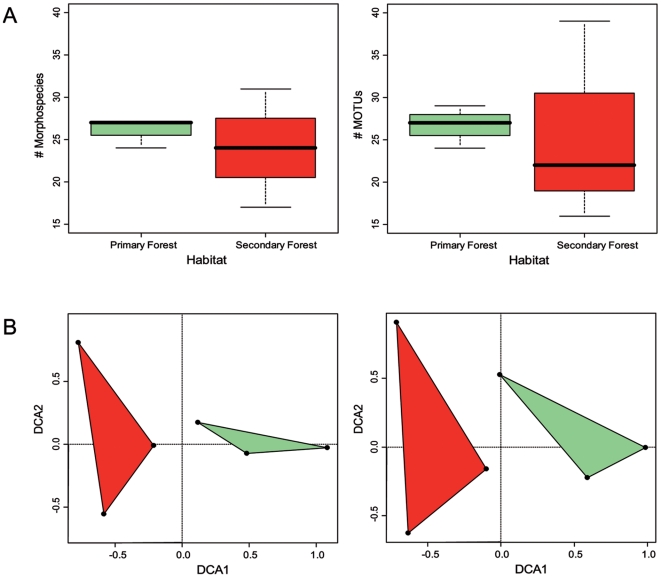
Comparison of diversity between primary and secondary forest. A. Species richness (number of morphospecies/MOTUs per transect) between primary forest (green) and secondary forest (red). B. Detrended correspondence analyses showing differences in species compositions of staphylinid beetle communities between primary and secondary forest, based on the morphological and the molecular data. Dots show positions of transects in ordination space spanned by DCA1 and DCA2. Spatial distance between transects indicates differences in species composition among transects. Green = Primary forest, red = Secondary forest.

When comparing beetle communities of primary and secondary forests, derived biodiversity patterns were highly consistent between the morphological and the molecular approach. Both approaches found no significant differences between secondary forests and primary forests in the richness (morphospecies richness: Welch-Two-Sample t-test, t = 0.48, df = 2.24, p = 0.67; MOTU richness: Welch-Two-Sample t-test, t = 0.14, df = 2.18, p = 0.90; Fig. 4A) and density of taxa (morphospecies density: Welch-Two-Sample t-test, t = 0, df = 3.55, p = 1; MOTU density: Welch-Two-Sample t-test, t = −0.02, df = 3.4, p = 0.98; see supplementary material Fig. S4). Interestingly, for all parameters the variance was estimated to be larger in secondary forest than in primary forest.

In contrast to species richness and species density, the species composition of staphylinid beetle communities differed significantly between primary and secondary forest habitats. This is indicated by the detrended correspondence analysis (DCA; see Fig. 4B) showing no overlap between the two habitats and a significant differentiation along the x-axis.

Seventy-eight percent (using the morphospecies data), respectively 77% (using the MOTU data) of the variation in the staphylinid community composition could be explained by the habitat (permutation test, p<0.001 for both data sets).

**Figure 5:**
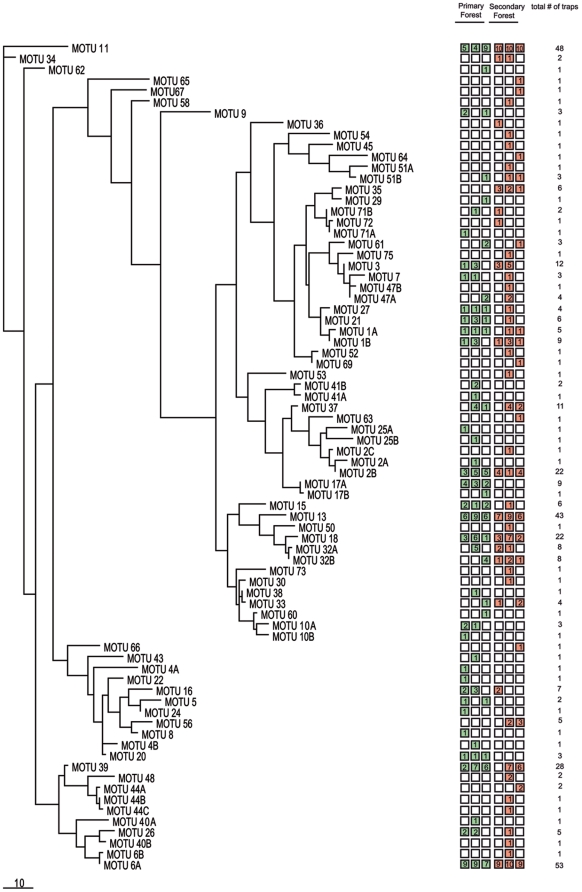
Neighbor-Joining-Tree of all MOTUs. Numbers indicate the number of traps in which the MOTU occurred.

These differences are produced by several morphospecies and MOTUs that have been collected exclusively or most abundantly in one habitat. Some of them occurred on two or even three transects (12 morphospecies and 8 MOTUs) and may represent species that are restricted to one of the two habitats (Fig. 5). It should be remarked that the Neighbor-Joining-Tree is not meant to represent a phylogeny but rather is to show the similarity of sequences graphically linked with the occurrence of the MOTUs on certain transects and possible habitat specialization.

## Discussion

### (a) Utility of DNA barcodes to reveal biodiversity parameters

Biodiversity assessments based on molecular sequence data may be a complement or an efficient alternative to traditional morphology-based approaches. However, the usefulness of the new methodology for inferring biodiversity patterns from non-microbial community samples which were collected using a standardized protocol as it is typically applied in ecological and conservation surveys and monitoring programs (e.g. [37], [49]), has rarely been tested to date (e.g. [6], [19], [20]). We found that the molecular and the morphological approach led to highly similar descriptions of biodiversity patterns: The similarity in total species richness and a high dissimilarity in species community composition between primary and secondary forests were revealed in a nearly identical way by both methods.

Smith et al. [19] and Smith and Fisher [6], who used MOTUs based on the COI barcoding marker and partly different nuclear markers to compare patterns of species diversity of ants within and between collection sites, also obtained similar results comparing morphological and molecular data. However, as a restricted number of specimens was analyzed from local communities (and procedures for specimen selection were not reported), it remained unknown if the used primer pairs and laboratory protocols would have been suitable for all species and specimens within the studied communities, a criterion which is of high importance for standardized biodiversity inventories. The use of universal primers is necessary for the analyses of samples whose sequences are not known *a priori*. A reliable marker is an important tool for the broad scale analyses of mixed environmental samples using next-generation sequencing technologies [50], [51], whose importance will probably increase in biodiversity research and conservation biology within the next years [51]. The D3-marker fulfills this criterion as it could be derived from nearly all individuals of the studied staphylinid community using one primer pair (CD3F/CD3R) and a single PCR protocol.

The high amplification success may be due to three advantages of the D3-marker, which are (i) the possibility to use primers that are universal enough to amplify a variety of species over a broad spectrum of genera, (ii) pre-amplification due to the tandem repeats of the ribosomal gene clusters, and (iii) its short length of only ∼180 bp, which allows an easy and efficient amplification even when using small amounts of tissue (as in our study, leading to a low concentration of DNA extracts) or in case of degraded DNA. This may be of practical value when using pitfall trapping (which is probably the most widely used collection method for soil insects) where degradation of DNA may happen due to dilution of ethanol because of evaporation or running-in rain water.

It should be acknowledged that the D3-marker in single cases may have failed to delimit some species. Therefore the estimated species numbers constitute a rather conservative result. The threshold of two, respectively three basepairs reduces the MOTU numbers and consequently changes biodiversity patterns but only slightly. In addition, it should be stressed that the marker sequences have not been used to delimit species accepted by taxonomists, but to distinguish meaningful biodiversity units as a surrogate for species. Even though not always species boundaries agree between the two approaches, the high similarity in detecting biodiversity patterns point out the heuristic value of the proposed MOTU approach.

Divergences between the morphological and the molecular approach resulted from splitting (two or more morphospecies are found in one MOTU) and lumping (two or more MOTUs are found for one morphospecies) of MOTUs. Splitting may be explained by sexual dimorphism or intraspecific morphological variability [52], [53], or on the other hand by a failure of the marker to resolve closely related species. In this context, the slightly higher number of morphospecies than MOTUs found per trap could be explained by the erroneous assignment of males and females of a sexually dimorphic species to two morphospecies. Lumpings may result from the occurrence of cryptic species (e.g. [54]), or the possibility that a difference of one basepair is within the range of intraspecific variation.

Although the separation of morphospecies was performed by an experienced beetle taxonomist, an erroneous assignment of specimens cannot completely be excluded. When handling high numbers of specimens and taxa, as typical for biodiversity studies in the tropics, it is hard even for an expert for a taxonomic group to keep an overview [41]. It should be added that Staphylinidae appear to be one of the more challenging families of Coleoptera, with partly quite high error rates in parataxomomic sorting [39], [55].

In cases where one MOTU includes individuals of different morphospecies with a suspiciously different appearance, or when morphospecies belong definitively to different genera, the possibility of contamination of samples or an erroneous assignment or denotation of sequences and of mounted specimens must be considered. This applies also to cases where specimens that have a high sequence divergence and therefore appear in different regions of the NJ-Tree, are assigned to the same morphospecies. Nevertheless, the number of contradictions was very low, probably affecting less than five taxa in total. They represent a specific error rate that cannot be excluded when a little known fauna is being studied and a quite large number of specimens is handled. Despite these theoretical and practical problems, the results of both methodological approaches show a high overlap in biodiversity patterns. This fact underscores their ability to detect true ecological patterns and is evidence for their methodological robustness.

In our study, DNA was extracted from single legs of mounted specimens and then amplified and sequenced in separate reactions. Therefore genetic data is explicitly linked to morphological specimens, which are stored in a zoological collection. A less time consuming approach would be to extract DNA from whole environmental samples (e.g. DNA from all specimens of a pitfall trap) and to process them using mass-amplification and mass-sequencing techniques [51]. However, using this approach the linkage between morphological and genetic information gets lost, which may hinder linking new information to the global taxonomic framework and the published ecological and evolutionary literature.

### (b) Conservation value of secondary forests for the staphylinid fauna inferred by D3-MOTUs

Secondary forests have reclaimed approximately 15% of the area of tropical rain forests cleared during the 1990s and are likely to be a dominant feature of tropical forest landscapes in the future. The extent to which they will be able to offset the loss of biodiversity from tropical deforestation is still being discussed [56]–[58]. The lack of significant differences in species richness between primary and secondary forests found in the present study must not lead to premature conclusions about the conservation value of secondary forests. The invasion of degraded areas by habitat generalists which are often of least conservation concern can mask the absence of habitat specialists by increasing the total species richness [59], [60]. Therefore, it is indispensable to analyze, in addition to total richness, changes in the community composition [49], [61]. Indeed, we found significant differences in the species communities of Staphylinidae comparing the two habitats and several morphospecies and MOTUs (and sometimes even MOTU clusters) seemed to be restricted to one of the habitats. Therefore, the present study suggests that for maintaining the natural staphylinid fauna in the area secondary forests are not an alternative to old growth rainforests. However, these questions could have been answered more precisely by a more thorough sampling with more comprehensive statistical analysis, but it should be noticed that the main aim of the present study is to point out the practical value of the proposed MOTU approach rather than answering the ecological questions per se.

## Supporting Information

Figure S1 **Map of Kakamega Forest.** Satellite map showing the location of the studied transects (kindly provided by G. Schaab).
(TIF)

Figure S2 **Species accumulation curve, showing the increase in the number of morphospecies (blue) and MOTUs (yellow) with increasing number of analyzed pitfall trap samples.** Coloured polygons indicate 95% confidence intervals.
(TIF)

Figure S3 **Mean species density (mean number of morphospecies/MOTUs per pitfall trap) on transects based on the morphological (blue) and the molecular genetic approach (yellow).**
(TIF)

Figure S4 **Mean species density (number of morphospecies/MOTUs per pitfall trap) between primary forest (green) and secondary forest (red).**
(TIF)

Table S1 **Studied transects.** Abbreviation, name, habitat, and coordinates.
(PDF)

Table S2 **List of Genbank accession numbers, voucher signatures and morphospecies names.**
(PDF)
