# Supplementary figures and images for: Testing a Short Nuclear Marker for Inferring Staphylinid Beetle Diversity in an African Tropical Rain Forest
Source: PLoS One. 2011 Mar 31;6(3):e18101. doi: 10.1371/journal.pone.0018101 (PMC3069053; doi:10.1371/journal.pone.0018101)

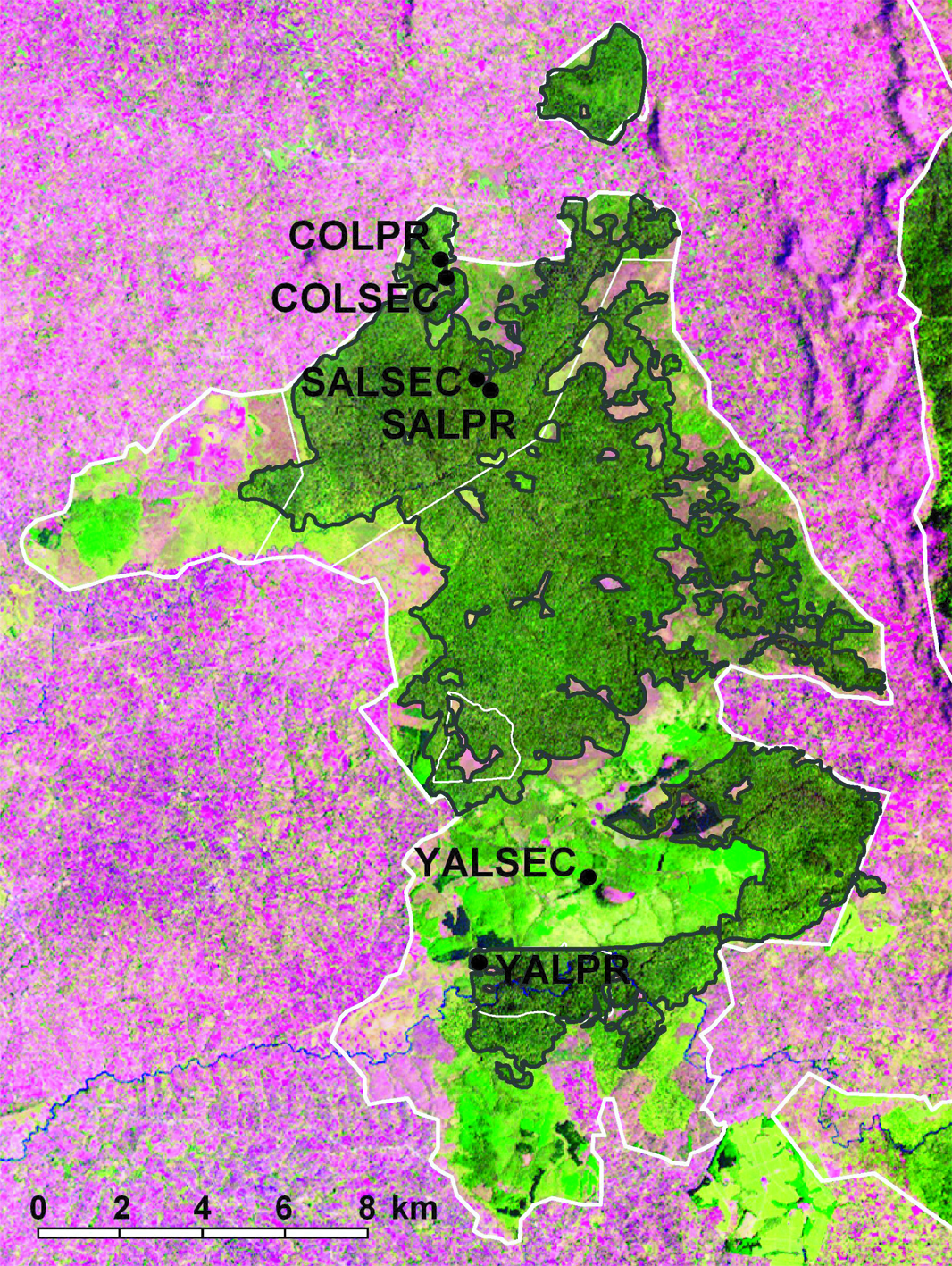

Supplement: Figure S1 — Map of Kakamega Forest. Satellite map showing the location of the studied transects (kindly provided by G. Schaab). (TIF) [file pone.0018101.s001.tif]

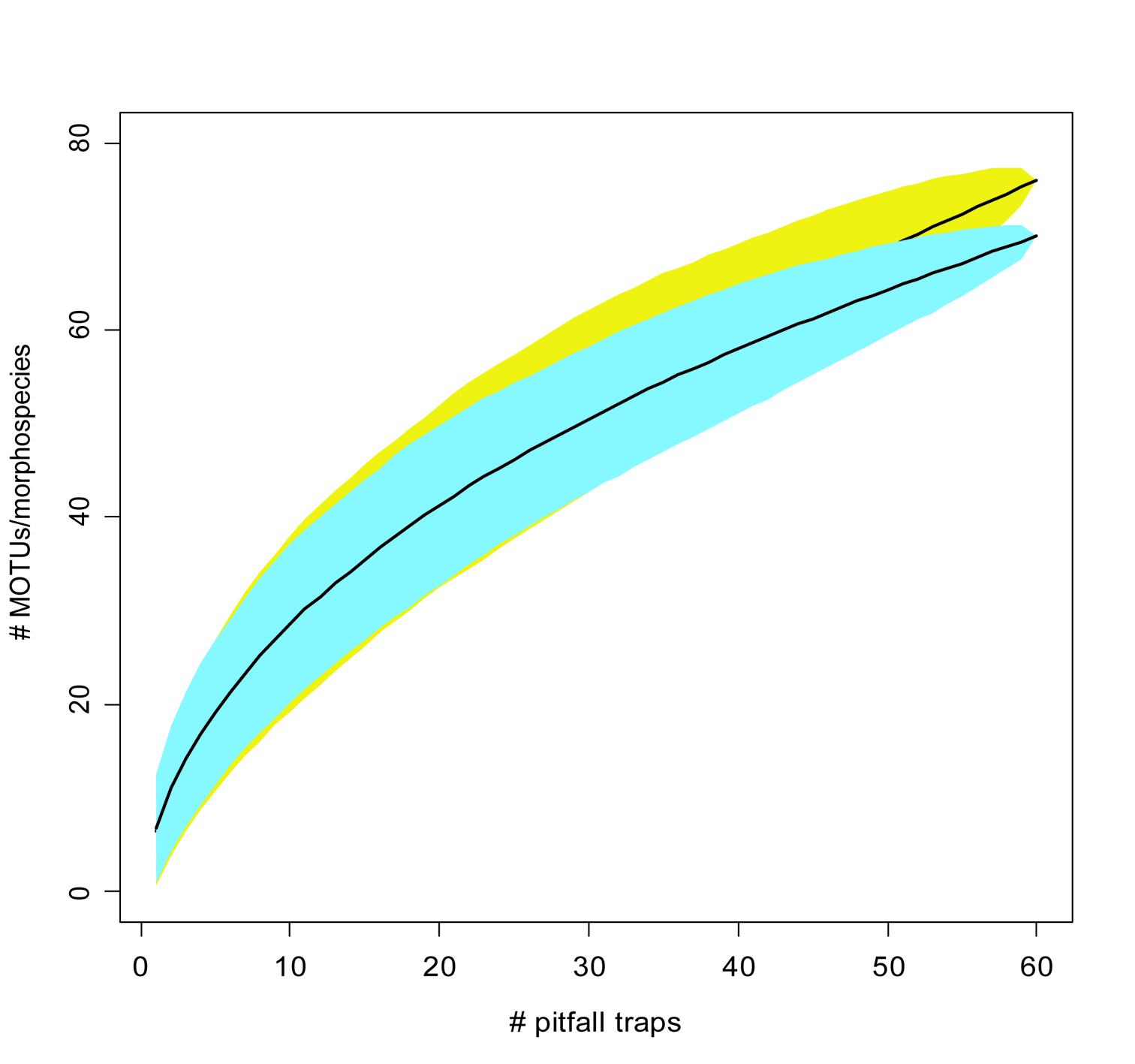

Supplement: Figure S2 — Species accumulation curve, showing the increase in the number of morphospecies (blue) and MOTUs (yellow) with increasing number of analyzed pitfall trap samples. Coloured polygons indicate 95% confidence intervals. (TIF) [file pone.0018101.s002.tif]

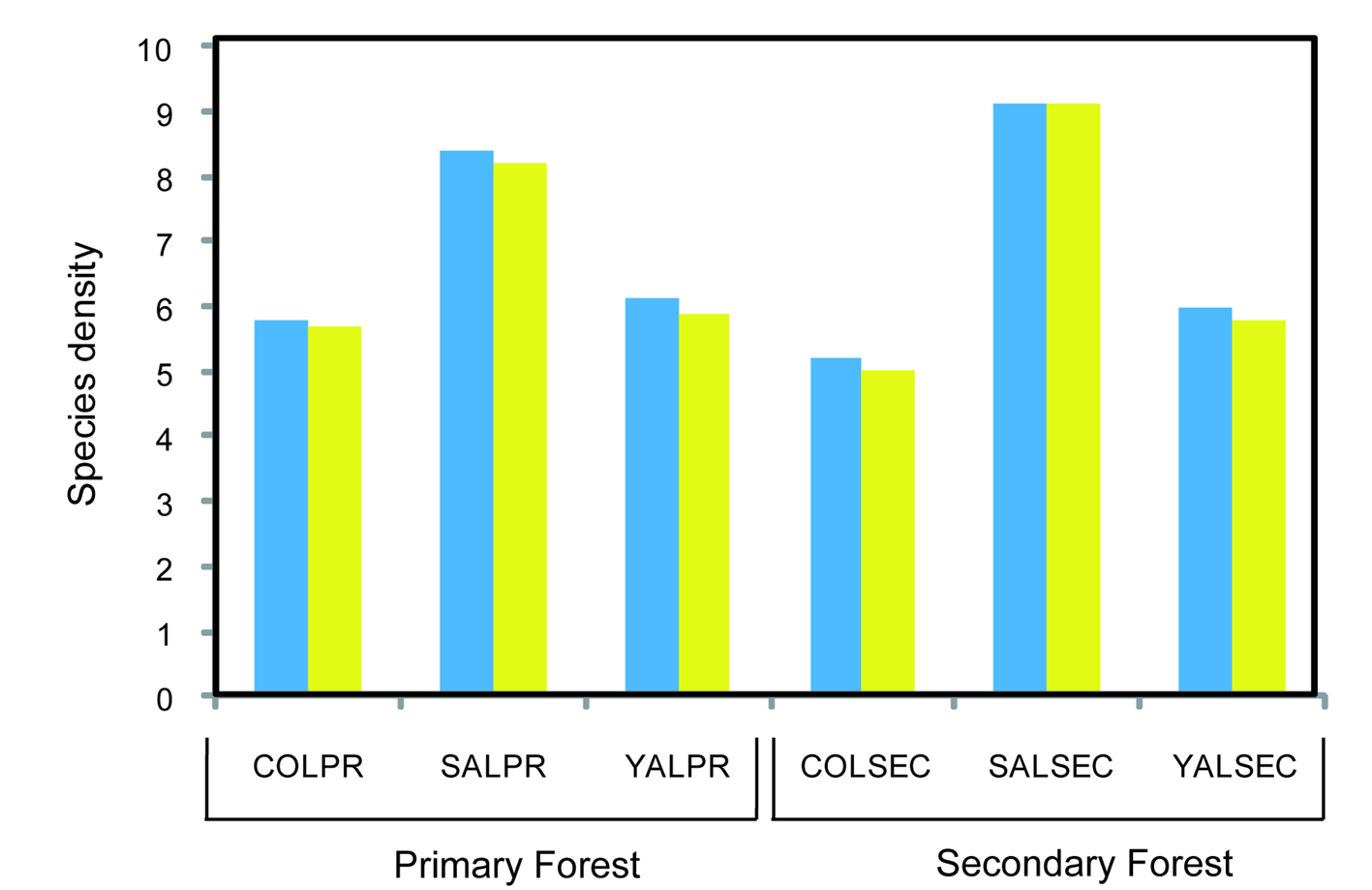

Supplement: Figure S3 — Mean species density (mean number of morphospecies/MOTUs per pitfall trap) on transects based on the morphological (blue) and the molecular genetic approach (yellow). (TIF) [file pone.0018101.s003.tif]

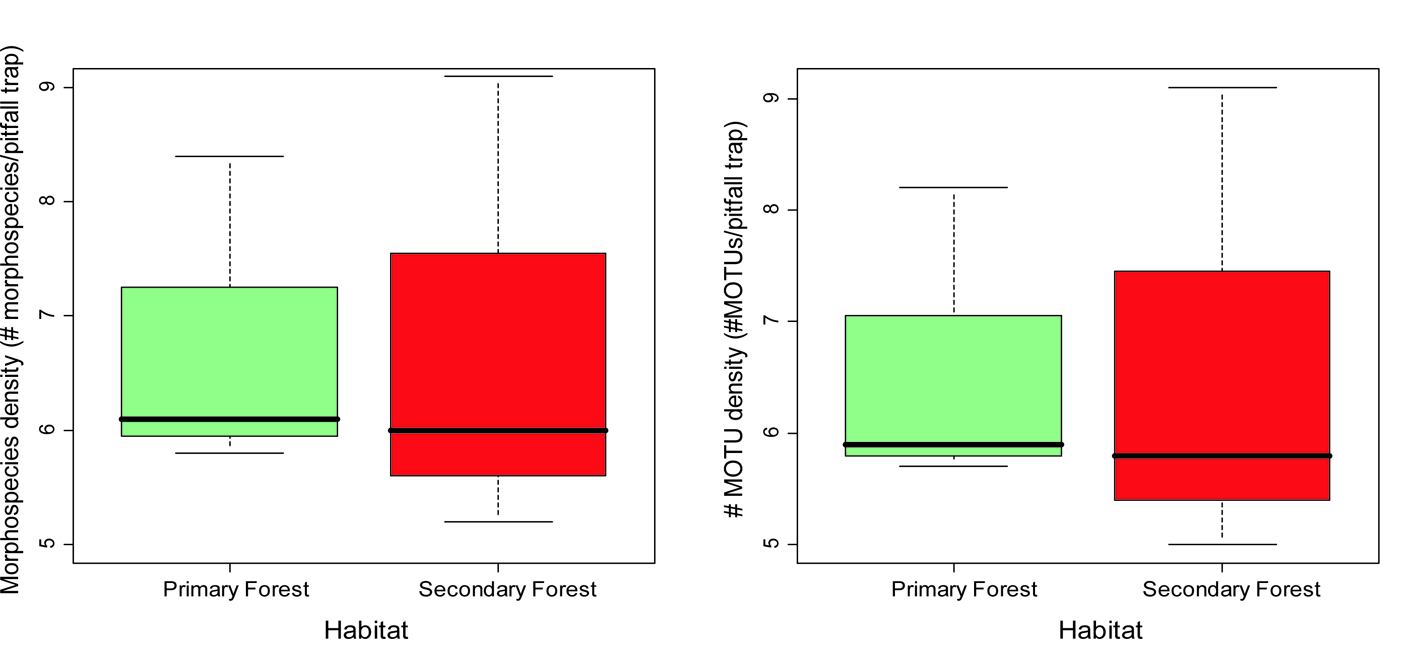

Supplement: Figure S4 — Mean species density (number of morphospecies/MOTUs per pitfall trap) between primary forest (green) and secondary forest (red). (TIF) [file pone.0018101.s004.tif]
