# Supplementary material for: Testing a Short Nuclear Marker for Inferring Staphylinid Beetle Diversity in an African Tropical Rain Forest
Source: PLoS One. 2011 Mar 31;6(3):e18101. doi: 10.1371/journal.pone.0018101 (PMC3069053; doi:10.1371/journal.pone.0018101)
Supplement: Table S1 — Studied transects. Abbreviation, name, habitat, and coordinates. (PDF) [file pone.0018101.s005.pdf]

Abbreviation	Transect name and number	Habitat	Longitude	Latitude
COLPR	Colobus Primary Forest	primary forest	E 34°51`36.10”	N 00°21`15.64”
COLSEC	Colobus Secondary Forest	secondary forest	E 34°51`40.87”	N 00°21`5.25”
SALPR	Salazar Primary Forest	primary forest	E 34°52`14.60”	N 00°19`36.00”
SALSEC	Salazar II Secondary Forest	secondary forest	E 34°52`2.80”	N 00°19`45.70”
YALPR	Yala Primary Forest	primary forest	E 34°52`6.00”	N 00°12`9.00”
YALSEC	Yala I Secondary Forest	secondary forest	E 34°53`29.70”	N 00°13`15.50”
