# Supplementary material for: Testing a Short Nuclear Marker for Inferring Staphylinid Beetle Diversity in an African Tropical Rain Forest
Source: PLoS One. 2011 Mar 31;6(3):e18101. doi: 10.1371/journal.pone.0018101 (PMC3069053; doi:10.1371/journal.pone.0018101)
Supplement: Table S2 — List of Genbank accession numbers, voucher signatures and morphospecies names. (PDF) [file pone.0018101.s006.pdf]

| Genbank accession number | Voucher Signature | Morphospecies name |
|---|---|---|
| HM583881 | ST_BT_BC00001 | Ocypus sp.2 |
| HM583882 | ST_BT_BC00002 | Atheta sp.3 |
| HM583883 | ST_BT_BC00161 | Atheta sp.6 |
| HM583884 | ST_BT_BC00004 | Rugilus sp.2 |
| HM583885 | ST_BT_BC00005 | Philonthus sp.2 |
| HM583886 | ST_BT_BC00006 | Zyras sp.6 |
| HM583887 | ST_BT_BC00007 | Hesperus sp.1 |
| HM583888 | ST_BT_BC00232 | Hesperus sp.2 |
| HM583889 | ST_BT_BC00008 | Oxytelus sp.5 |
| HM583890 | ST_BT_BC00009 | Zyras sp.2 |
| HM583891 | ST_BT_BC00010 | Oxytelus sp.6 |
| HM583892 | ST_BT_BC00011 | Aleochara sp. |
| HM583893 | ST_BT_BC00020 | Zyras sp.4 |
| HM583894 | ST_BT_BC00021 | Anotylus sp.2 |
| HM583895 | ST_BT_BC00052 | Oxypoda sp.1 |
| HM583896 | ST_BT_BC00142 | Oxypoda sp.2 |
| HM583897 | ST_BT_BC00029 | Oxytelus sp.3 |
| HM583898 | ST_BT_BC00305 | Oxytelus sp.4 |
| HM583899 | ST_BT_BC00033 | Lathrobium |
| HM583900 | ST_BT_BC00289 | Ocypus sp.3 |
| HM583901 | ST_BT_BC00035 | Zyras sp.3 |
| HM583902 | ST_BT_BC00042 | Ocypus sp.1 |
| HM583903 | ST_BT_BC00047 | Anotylus sp.2 |
| HM583904 | ST_BT_BC00050 | Zyras sp.1 |
| HM583905 | ST_BT_BC00051 | Medon sp.1 |
| HM583906 | ST_BT_BC00091 | Oxytelus glabratus |
| HM583907 | ST_BT_BC00092 | Megarthrus sp. |
| HM583908 | ST_BT_BC00257 | Leptusa sp.1 |
| HM583909 | ST_BT_BC00100 | Leptusa sp.2 |
| HM583910 | ST_BT_BC00109 | Oxytelus glabratus |
| HM583911 | ST_BT_BC00111 | Osorius sp.1 |
| HM583912 | ST_BT_BC00125 | Rugilus sp.3 |
| HM583913 | ST_BT_BC00130 | Anotylus sp.3 |
| HM583914 | ST_BT_BC00135 | Medon sp.1 |
| HM583915 | ST_BT_BC00140 | Oxypoda sp.3 |
| HM583916 | ST_BT_BC00014 | Atheta sp.1 |
| HM583917 | ST_BT_BC00132 | Atheta sp.2 |
| HM583918 | ST_BT_BC00153 | Rugilus sp.2 |
| HM583919 | ST_BT_BC00156 | Zyras sp.4 |
| HM583920 | ST_BT_BC00158 | Osorius sp.2 |
| HM583921 | ST_BT_BC00172 | Coprophilus sp. |
| HM583922 | ST_BT_BC00180 | Ososrius sp.2 |
| HM583923 | ST_BT_BC00036 | Zyras sp.7 |
| HM583924 | ST_BT_BC00199 | Zyras sp.8 |
| HM583925 | ST_BT_BC00208 | Rugilus sp.1 |
| HM583926 | ST_BT_BC00258 | Atheta sp.5 |

| | | |
|---|---|---|
| HM583927 | ST_BT_BC00229 | Atheta sp.7 |
| HM583928 | ST_BT_BC00234 | Philonthus sp.1 |
| HM583929 | ST_BT_BC00239 | Atheta sp.7 |
| HM583930 | ST_BT_BC00240 | Gyrohypnus |
| HM583931 | ST_BT_BC00247 | Rabigus sp.1 |
| HM583932 | ST_BT_BC00068 | Ocypus sp.4 |
| HM583933 | ST_BT_BC00105 | Ocypus sp.5 |
| HM583934 | ST_BT_BC00254 | Oxytelus sp.2 |
| HM583935 | ST_BT_BC00255 | Xantholinus sp.2 |
| HM583936 | ST_BT_BC00256 | Anotylus sp.5 |
| HM583937 | ST_BT_BC00268 | Anotylus sp.2 |
| HM583938 | ST_BT_BC00274 | Cryptobium sp. |
| HM583939 | ST_BT_BC00275 | Philonthus sp.5 |
| HM583940 | ST_BT_BC00276 | Xantholinus sp.2 |
| HM583941 | ST_BT_BC00017 | Oxytelus sp.1 |
| HM583942 | ST_BT_BC00030 | Anotylus sp.4 |
| HM583943 | ST_BT_BC00066 | Oxytelus sp.2 |
| HM583944 | ST_BT_BC00283 | Atheta sp.2 |
| HM583945 | ST_BT_BC00295 | Rugilus sp.3 |
| HM583946 | ST_BT_BC00296 | Philonthus sp.2 |
| HM583947 | ST_BT_BC00291 | Falagria sp. |
| HM583948 | ST_BT_BC00303 | Philonthus sp.1 |
| HM583949 | ST_BT_BC00308 | Atheta sp.3 |
| HM583950 | ST_BT_BC00315 | Ischnopoda sp. |
| HM583951 | ST_BT_BC00003 | Zyras sp.5 |
| HM583952 | ST_BT_BC00332 | Rugilus sp.1 |
| HM583953 | ST_BT_BC00334 | Ocypus sp.4 |
| HM583954 | ST_BT_BC00344 | Anotylus sp.1 |
| HM583955 | ST_BT_BC00349 | Philonthus sp.4 |
| HM583956 | ST_BT_BC00457 | Philonthus sp.3 |
| HM583957 | ST_BT_BC00378 | Atheta sp.4 |
| HM583958 | ST_BT_BC00407 | Sunius sp. |
| HM583959 | ST_BT_BC00425 | Xantholinus sp.1 |
| HM583960 | ST_BT_BC00427 | Euaesthetus sp. |
| HM583961 | ST_BT_BC00428 | Bolitochara sp. |
| HM583962 | ST_BT_BC00429 | Atheta sp.8 |
| HM583963 | ST_BT_BC00451 | Rabigus sp.2 |
| HM583964 | ST_BT_BC00431 | Atheta sp.7 |
| HM583965 | ST_BT_BCXL1 | Platydracus sp.1 |
| HM583966 | ST_BT_BCXL2 | Platydracus sp.2 |
| HM583967 | ST_BT_BCXL3 | Platydracus sp.1 |
